# Acute Pain Management in a Multi-site Trauma Patient

**DOI:** 10.7759/cureus.61596

**Published:** 2024-06-03

**Authors:** Michael Ayad, Malcolm Lee, Jose L Diz Ferre, Lori Ann Oliver, Sabry Ayad

**Affiliations:** 1 Outcomes Research Consortium, Lake Erie College of Osteopathic Medicine, Cleveland, USA; 2 Outcomes Research Consortium, Ohio University Heritage College of Osteopathic Medicine, Cleveland, USA; 3 Outcomes Research, Cleveland Clinic, Cleveland, USA; 4 Anesthesiology, Cleveland Clinic Fairview Hospital, Cleveland, USA; 5 Anesthesiology, Cleveland Clinic, Cleveland, USA

**Keywords:** acute pain management, complex trauma mva (motor vehicle accident), paravertebral block (pvb), opioid-sparing analgesia, multimodal pain management, substance use disorder (sud), adductor canal block (acb), popliteal nerve block, ultrasound guided nerve block, local anesthetic systemic toxicity (last)

## Abstract

Pain management is often difficult in the setting of multi-site trauma such as that caused by motor vehicle accidents (MVA), which is especially compounded in the setting of polysubstance abuse. This often results in patients with poor pain tolerance requiring escalating doses of opioid therapy, which creates a vicious cycle. The use of peripheral nerve blocks (PNB) has been shown to decrease overall opioid consumption and can be used effectively to manage postoperative pain in this patient population. Our case report aims to highlight the importance of PNBs as part of a multimodal approach to pain management in patients with polytrauma in the setting of polysubstance abuse.

## Introduction

The clinical management of multi-site trauma, such as that caused by a motor vehicle accident (MVA), can be highly challenging, especially in patients with polysubstance abuse. These patients often present with poorly controlled pain, requiring escalating doses of opioids, which creates a vicious cycle of dependency and tolerance. Effective pain management in this population is further complicated by factors such as altered pain thresholds, potential drug interactions, and the risk of withdrawal symptoms.

Several pain management options are available for such patients, including systemic analgesics, regional anesthesia techniques, and multimodal analgesia strategies. While systemic analgesics, such as opioids, are commonly used, they can lead to increased tolerance and dependence, particularly in patients with a history of substance abuse. Non-opioid analgesics, including acetaminophen and nonsteroidal anti-inflammatory drugs (NSAIDs), can be beneficial but may not provide sufficient pain relief on their own. Regional anesthesia techniques, such as epidural analgesia and peripheral nerve blocks (PNBs), offer significant advantages by providing targeted pain relief with reduced systemic side effects. They have been shown to decrease overall opioid consumption and can be used effectively to manage postoperative pain [1]. In this report, we aim to highlight the importance of PNBs as part of a multimodal approach to pain management in patients with polytrauma in the setting of polysubstance abuse.

## Case presentation

The patient was a 26-year-old female with a BMI of 33 (77.1 kg/1.52 m^2^) and a past medical history significant for supraventricular tachycardia, postpartum depression, and drug abuse. She arrived as trauma level 2 via emergency medical services (EMS) after an MVA with airbag deployment. Upon arrival at the emergency department, her vital signs showed a temperature of 37.2 °C (98.9 °F), a blood pressure of 113/86 mmHg, a pulse of 71 bpm, a respiratory rate of 17 bpm, and a saturation of 99% on room air. During the secondary survey, the physical exam was significant for a large laceration extending over the left lateral knee joint and positive for swelling and tenderness extending to the ankle of the right lower extremity. The overall pain score was 10/10 at rest.

**Figure 1:**
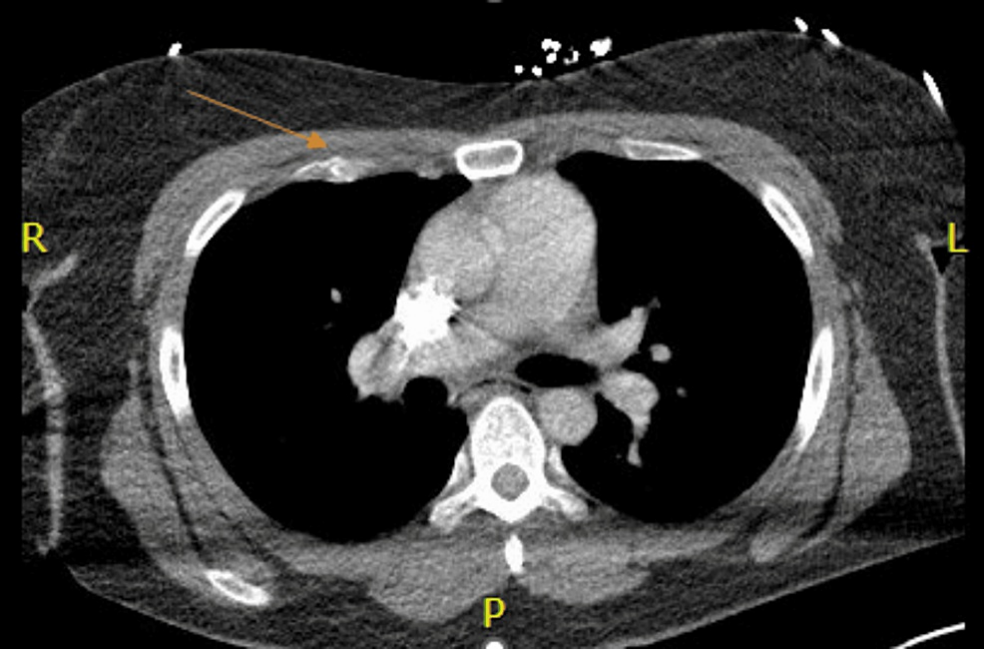
CT axial scan of the chest with intravenous contrast - image 1 The orange arrow shows nondisplaced anterior rib fractures CT: computed tomography

**Figure 2:**
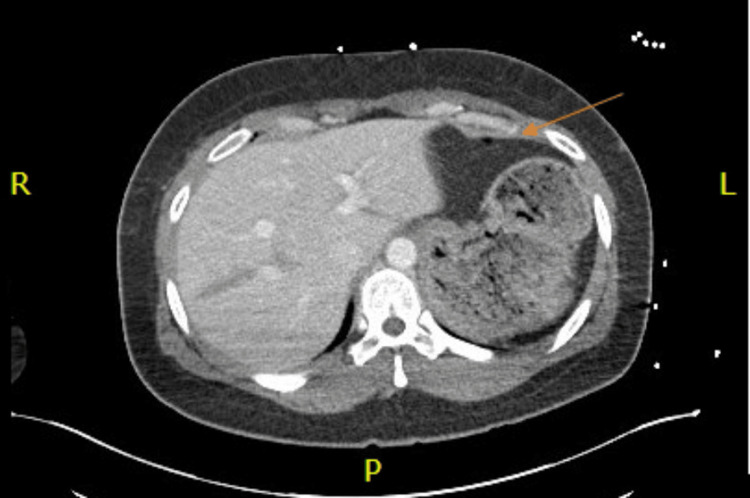
CT axial scan of the chest with intravenous contrast - image 2 The orange arrow shows grade III traumatic liver injury consisting of a laceration in the left lobe extending at least 7 cm with additional parenchymal contusion about the site of laceration without evidence of active bleeding CT: computed tomography

**Figure 3:**
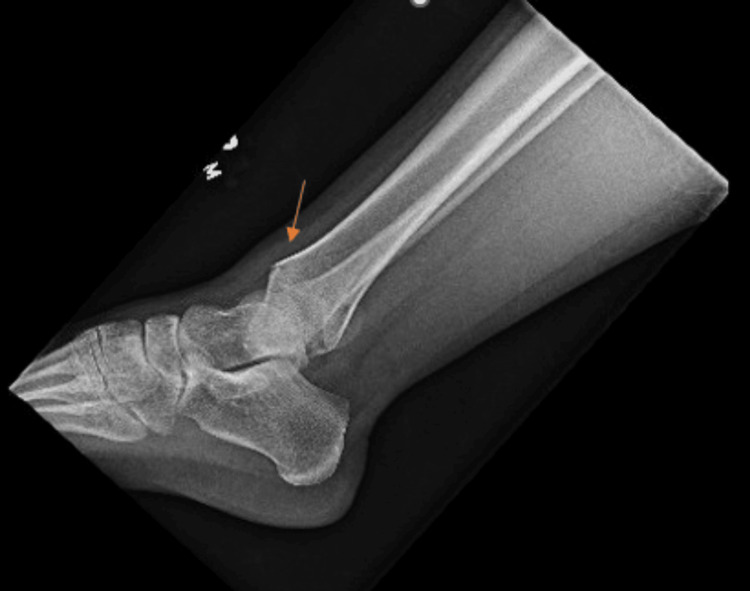
Lateral radiograph of the ankle X-ray series The orange arrow shows a nondisplaced fracture of the distal tibial metadiaphysis with extension to the articular surface laterally

Toxicology screen was positive for amphetamines, cocaine, and cannabis. Chest CT showed multiple nondisplaced anterior rib fractures, trace bilateral apical pneumothoraces, and a nondisplaced fracture involving the anterior cortex of the mid-sternal body (Figure 1). CT of the abdomen and pelvis showed additional grade III traumatic liver injury comprising a laceration in the mid-aspect of the left lobe extending from the lateral capsule at least 7 cm with an additional parenchymal contusion about the site of laceration (Figure 2). Also, heterogeneous enlargement of the right adrenal gland was consistent with traumatic adrenal hematoma, and a small amount of hemoperitoneum in the pelvis was seen. The right lower extremity X-ray showed a nondisplaced fracture of the distal tibial metaphysis with extension to the articular surface laterally, and a mildly displaced posterior right tibial malleolar fracture (Figure 3).

**Figure 4:**
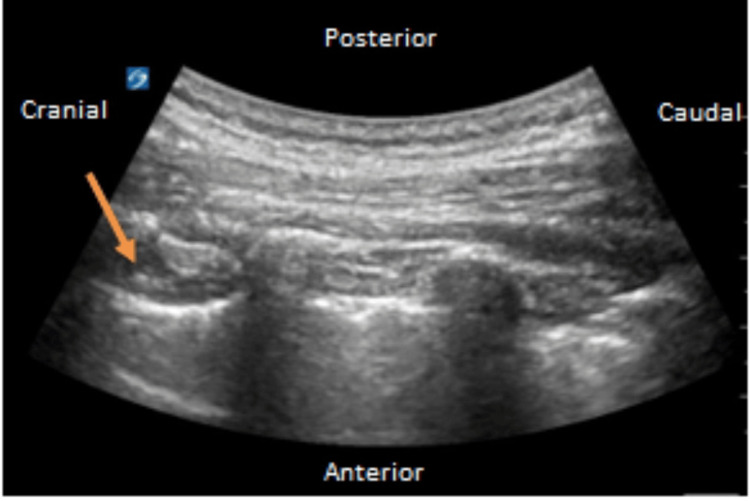
Ultrasound-guided paravertebral catheter placement with convex probe The catheter was at a skin depth of 11 cm and the second catheter was at a skin depth was 10 cm. Needle insertion depth was 5 cm and 4.5 cm. Orange arrow: the in-plane tip of the needle. Good pleural depression was visualized following an injection of saline only. Pleural sliding was present pre and post-procedure with negative aspirate

The patient was transferred to the ICU for the management of multiple rib fractures; the Acute Pain Management Service (APMS) was consulted and they decided to place bilateral T4 paravertebral block (PVB) catheters with ultrasound guidance to aid in pain control and minimization of opioids in the ICU (Figure 4) [2]. The blocks were placed with the patient in the sitting position followed by an intermittent dosing regimen of ropivacaine 0.2% at 6 mL (24 mg) every 30 minutes. The multimodal approach included diclofenac and lidocaine patches as well as intravenous (IV) hydromorphone 0.4 mg, oral oxycodone 5 mg as needed, and cyclobenzaprine 750 mg for muscle relaxation. The pain was adequately controlled.

**Figure 5:**
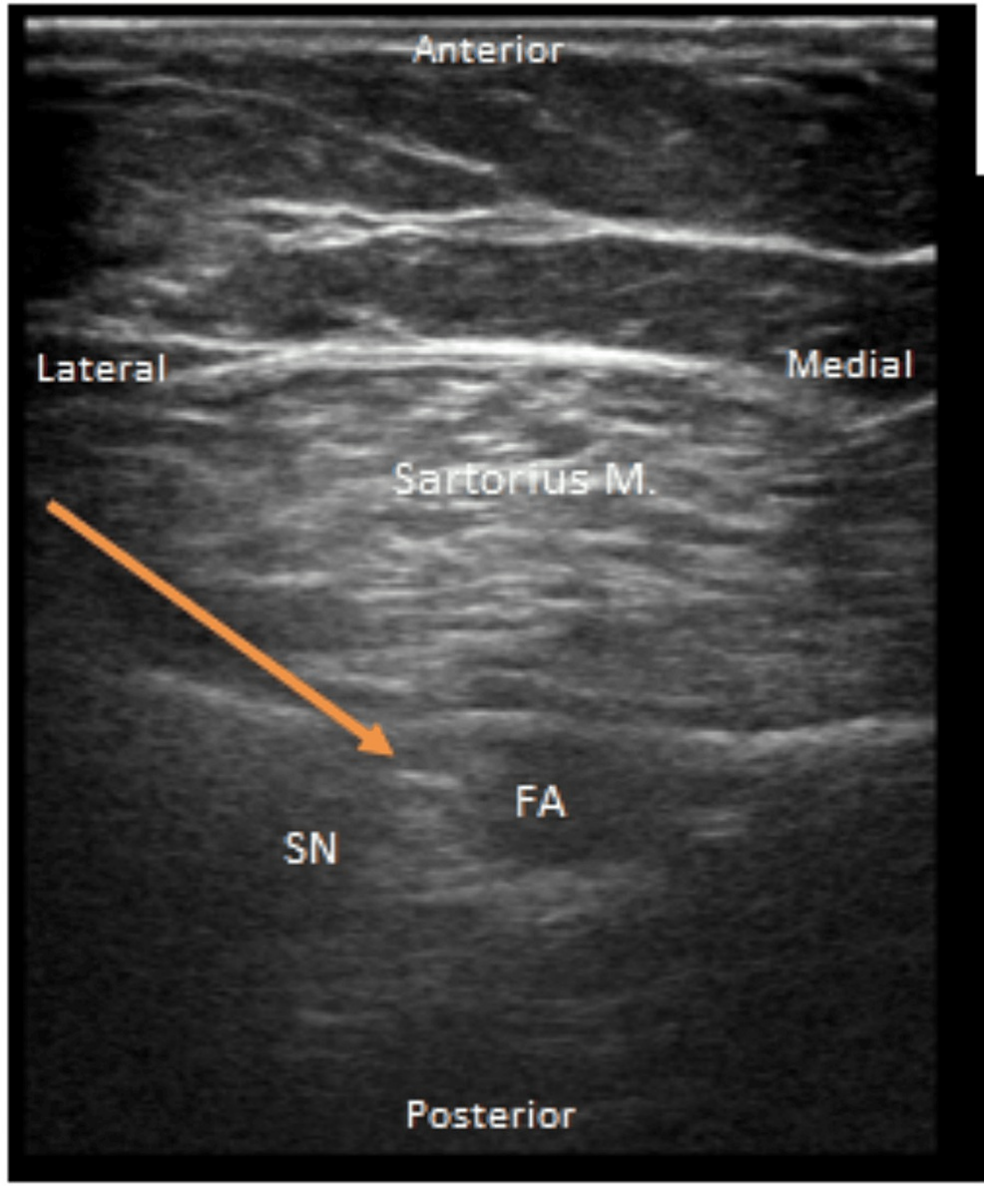
Ultrasound-guided right adductor canal catheter placement with linear probe The catheter was at a skin depth of 10 cm and the needle was inserted at a depth of 6 cm. Tuohy 18 g 10 cm needles with 20 g catheters and injection assessment showed negative aspiration, and no paresthesia on injection. Orange arrow: the in-plane tip of the needle SN: saphenous nerve; FA: femoral artery

**Figure 6:**
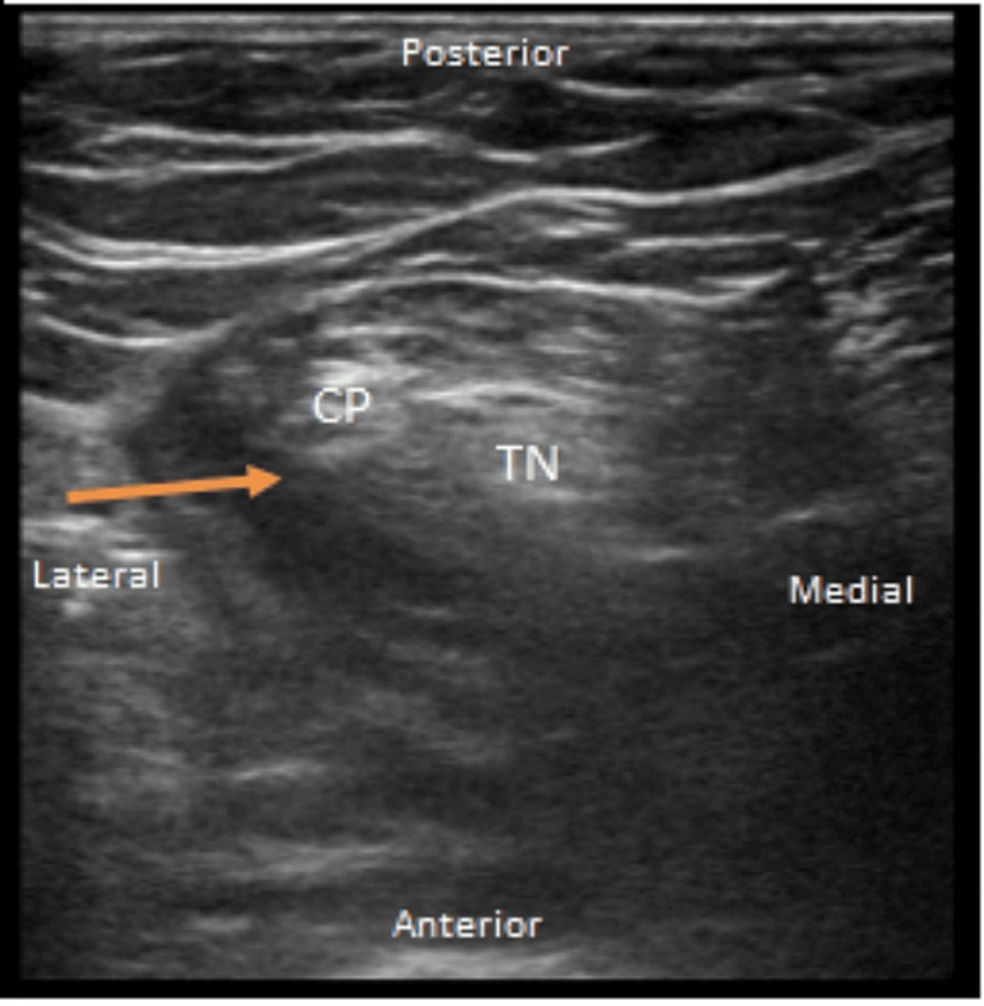
Ultrasound-guided right popliteal catheter placement with linear probe The catheter was at a skin depth of 6 cm and the needle insertion depth was 6 cm. Tuohy 18 g 10 cm needles with 20 g catheters and injection assessment showed negative aspiration, and no paresthesia on injection. Orange arrow: the in-plane tip of the needle CP: common peroneal; TN: tibial nerve

On day three, the patient underwent open reduction and internal fixation (ORIF) of a right pilon fracture under general anesthesia. The patient was extubated and transferred to the PACU and had poorly controlled pain requiring escalating doses of IV opioids. The APMS was consulted for rescue PNBs. Adductor and popliteal catheters using 20 mL (40 mg) of 0.2% ropivacaine were used in the PACU [3]. The sites were prepped with 2% chlorhexidine gluconate/70% isopropyl alcohol formulation and the skin was completely dried before the procedure. Ultrasound guidance was used, and the images were recorded in the chart as well (Figures 5-6). The APMS started both infusions of ropivacaine 0.2% at 6 ml (24 mg) every 30 minutes. The patient's pain score decreased from 10 at rest and ambulation to 0 at rest and 4 on ambulation. The following day, the concentrations were decreased to ropivacaine 0.1% to prevent local anesthetic systemic toxicity (LAST). On postoperative day two, PVB catheters were removed with no increase in pain scores. On postoperative day four, lower extremity nerve block catheters were also removed, and the patient was subsequently discharged.

## Discussion

The use of multiple PNB catheters with local anesthetic infusions can be a cause for concern due to the potential risk of LAST [4]; it is a rare but potentially life-threatening complication of regional anesthesia that occurs when local anesthetic agents are absorbed into the systemic circulation in amounts that exceed toxic thresholds. It can lead to central nervous system and cardiovascular toxicity, with manifestations ranging from mild to severe symptoms, including dizziness, tinnitus, seizures, cardiovascular collapse, and even death. Epidural analgesia, while effective, carries risks such as hypotension, urinary retention, and potential for epidural hematoma, especially in patients receiving anticoagulation therapy. In contrast, PNBs can provide targeted analgesia with a potentially lower risk of systemic complications. Given the extent of our patient’s injuries, she was immobilized with limited PT and was receiving anticoagulation, which was a relative contraindication for epidural placement.

PVBs or other peripheral nerve blocks such as erector spinae plane (ESP) blocks [5] are increasingly used to manage rib fracture pain, as they have been shown to have comparable pain relief to epidurals with a better safety profile [6]. Serratus anterior plane (SAB) blocks can also be used for the management of rib fracture pain for patients in whom the placement of PVB, ESP, and epidural placement are contraindicated. In addition, SABs have been also utilized in patients who cannot be moved to access the posterior aspect due to various reasons such as polytrauma [7].

In managing our patient, we adhered to established safe-dosing guidelines for ropivacaine, which recommend a dose of 2-3 mg/kg/day. We used the lowest effective dose of local anesthetic and continuously monitored for early signs of toxicity [8]. The placement of four catheters involved meticulous monitoring of early signs of toxicity. We carefully adjusted each infusion from all catheters to use the lowest effective dose of local anesthetic. Continuous monitoring of the patient's vital signs and level of sedation is also essential, particularly in patients with acute kidney or liver insufficiency, malabsorptive syndromes, or low-binding proteins since they are at a higher risk of LAST.

Our patient was young and had normal kidney function. However, we still prioritized continuous monitoring to promptly detect any early signs of toxicity and mitigate this potential complication. Our patient’s average vital signs were consistently stable throughout the course of her admission. The combination of multimodal analgesia and nerve blocks resulted in successful pain control and rehabilitation. She showed positive outcomes from inpatient care, including ambulation and stair-climbing before her discharge home on day seven.

## Conclusions

This case report highlights the need for a holistic approach to managing polytrauma patients in the setting of substance abuse and high opioid requirements. The use of multiple PNBs provided effective pain control and facilitated early recovery while minimizing the risks associated with systemic local anesthetic toxicity. This report also underscores the importance of a comprehensive pain management strategy in complex trauma cases due to the high level of pain at presentation. The patient should be monitored at all times for the signs and symptoms of LAST. A high level of vigilance among the providers regarding the recognition of LAST is critical for optimizing patient outcomes.
